# (2-Amino-3-nitro­benzoato-κ*O*)triphenyl­tin(IV)

**DOI:** 10.1107/S160053681101244X

**Published:** 2011-04-13

**Authors:** Yip-Foo Win, Chen-Shang Choong, Mei-Hsuan Heng, Ching Kheng Quah, Hoong-Kun Fun

**Affiliations:** a Department of Chemical Science, Faculty of Science, Universiti Tunku Abdul Rahman, 31900 Kampar, Perak, Malaysia; b X-ray Crystallography Unit, School of Physics, Universiti Sains Malaysia, 11800 USM, Penang, Malaysia

## Abstract

The asymmetric unit of the title compound, [Sn(C_6_H_5_)_3_(C_7_H_5_N_2_O_4_)], consists of two independent mol­ecules. In each mol­ecule, the four-coordinated Sn^IV^ atom exists in a distorted tetra­hedral geometry and two intra­molecular N—H⋯O hydrogen bonds with *S*(6) ring motifs are present. In one mol­ecule, the benzene ring of the 2-amino-3-nitro­benzoate ligand makes dihedral angles of 42.74 (11), 89.66 (13) and 53.04 (10)° with the three phenyl rings. The corresponding dihedral angles for the other mol­ecule are 6.29 (11), 66.55 (11) and 62.33 (10)°. In the crystal, a weak inter­molecular C—H⋯π inter­action and a π–π stacking inter­action with a centroid–centroid distance of 3.5877 (12) Å are observed.

## Related literature

For general background to and the coordination environment of the title complex, see: Yeap & Teoh (2003 ▶); Win *et al.* (2007 ▶, 2008 ▶, 2010 ▶). For bond-length data, see: Allen *et al.* (1987 ▶). For hydrogen-bond motifs, see: Bernstein *et al.* (1995 ▶). 
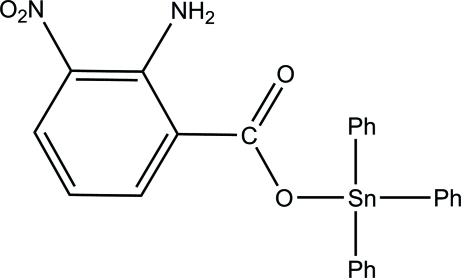


## Experimental

### 

#### Crystal data

[Sn(C_6_H_5_)_3_(C_7_H_5_N_2_O_4_)]
*M* *_r_* = 531.12
Triclinic, 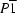

*a* = 11.2836 (1) Å
*b* = 14.9600 (2) Å
*c* = 15.1828 (3) Å
α = 109.257 (1)°
β = 98.503 (1)°
γ = 105.743 (1)°
*V* = 2247.89 (6) Å^3^
*Z* = 4
Mo *K*α radiation
μ = 1.17 mm^−1^
*T* = 296 K
0.44 × 0.32 × 0.19 mm

#### Data collection

Bruker SMART APEXII CCD area-detector diffractometer
Absorption correction: multi-scan (*SADABS*; Bruker, 2009 ▶) *T*_min_ = 0.628, *T*_max_ = 0.810
69259 measured reflections
18711 independent reflections
12707 reflections with *I* > 2σ(*I*)
*R*_int_ = 0.027

#### Refinement

*R*[*F* ^2^ > 2σ(*F* ^2^)] = 0.032
*wR*(*F* ^2^) = 0.080
*S* = 1.01
18711 reflections
593 parameters
H atoms treated by a mixture of independent and constrained refinement
Δρ_max_ = 0.70 e Å^−3^
Δρ_min_ = −0.42 e Å^−3^

### 

Data collection: *APEX2* (Bruker, 2009 ▶); cell refinement: *SAINT* (Bruker, 2009 ▶); data reduction: *SAINT*; program(s) used to solve structure: *SHELXTL* (Sheldrick, 2008 ▶); program(s) used to refine structure: *SHELXTL*; molecular graphics: *SHELXTL*; software used to prepare material for publication: *SHELXTL* and *PLATON* (Spek, 2009 ▶).

## Supplementary Material

Crystal structure: contains datablocks global, I. DOI: 10.1107/S160053681101244X/is2696sup1.cif

Structure factors: contains datablocks I. DOI: 10.1107/S160053681101244X/is2696Isup2.hkl

Additional supplementary materials:  crystallographic information; 3D view; checkCIF report

## Figures and Tables

**Table 1:** Hydrogen-bond geometry (Å, °) *Cg*1 is the centroid of the C1*A*–C6*A* phenyl ring.

*D*—H⋯*A*	*D*—H	H⋯*A*	*D*⋯*A*	*D*—H⋯*A*
N1*A*—H1*NA*⋯O3*A*	0.84 (3)	2.02 (3)	2.632 (3)	129 (2)
N1*A*—H2*NA*⋯O2*A*	0.84 (3)	1.99 (3)	2.671 (3)	138 (2)
N1*B*—H1*NB*⋯O2*B*	0.84 (2)	1.98 (3)	2.643 (3)	135 (2)
N1*B*—H2*NB*⋯O3*B*	0.83 (2)	1.96 (2)	2.607 (3)	135.3 (19)
C15*B*—H15*B*⋯*Cg*1^i^	0.93	2.84	3.596 (3)	139

Symmetry code: (i) 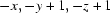.

**Fig. 1.**
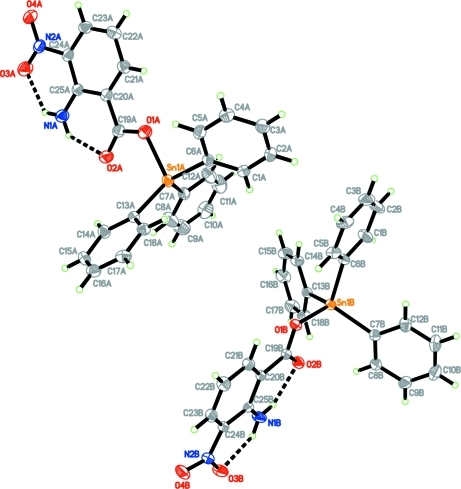
The asymmetric unit of the title compound, showing 20% probability displacement ellipsoids for non-H atoms.

**Fig. 2.**
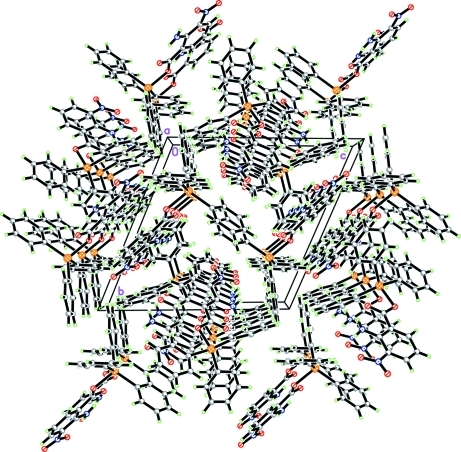
Packing diagram of the title compound, viewed along the a axis.

## supplementary crystallographic information

### Comment

Commonly, triphenyltin(IV) carboxylate complexes exist as a monomeric and polymeric structures (Yeap &Teoh, 2003; Win *et al.*, 2007, 2008, 2010). For monomeric structures, the tin(IV) moiety could be either four- or five-coordinated. However, for polymeric structures, the tin(IV) moiety normally exist in five-coordinated (Win *et al.*, 2010). The title complex is found to be similar to the reported structure of (2-amino-5-nitrobenzoato)triphenyltin(IV) (Win *et al.*, 2007) with the exception that the nitro group is in a different position at the benzoate moiety in this study.

The asymmetric unit contains two independent molecules (Fig. 1), *A* and *B*. In each molecule, the four-coordinate tin atom (Sn1A/Sn1B) exists in a distorted tetrahedral geometry, formed by a monodentate carboxylate group and three phenyl rings. The molecular structure is stabilized by intramolecular N1A—H1NA···O3A, N1A—H2NA···O2A, N1B—H1NB···O2B and N1B—H2NB···O3B hydrogen bonds (Table 1) which generate *S*(6) ring motifs (Fig. 1; Bernstein *et al.*, 1995). Bond lengths (Allen *et al.*, 1987) and angles are within normal ranges. In molecule *A*, the phenyl ring (C20A–C25A) of 2-amino-3-nitrobenzoate moiety makes dihedral angles of 42.74 (11), 89.66 (13) and 53.04 (10)° with respect to the three phenyl rings (C1A–C6A, C7A–C12A and C13A–C18A). The corresponding dihedral angles for molecule *B* are 6.29 (11), 66.55 (11) and 62.33 (10)°.

In the crystal (Fig. 2), a weak intermolecular C—H···π interaction (Table 1) and a π–π stacking interaction between two phenyl rings (C20B–C25B, centroid *Cg*2), with a *Cg*2···*Cg*2 distance of 3.5877 (12) Å are observed. No significant intermolecular hydrogen bond is observed.

### Experimental

The title complex was obtained by heating under reflux a 1:1 molar mixture of triphenyltin(IV) hydroxide (0.73 g, 2 mmol) and 2-amino-3-nitrobenzoic acid (0.36 g, 2 mmol) in methanol (60 mL) for 2 h. A clear yellow transparent solution was separated by filtration and kept in a bottle. After a few days, yellow crystals (0.46 g, 86.0 % yield) were collected (*m.p.* 155.0–156.0 °C). Analysis for C_25_H_20_N_2_O_4_Sn: C 56.72, H 3.73, N 5.24%. Calculated for C_25_H_20_N_2_O_4_Sn: C 56.53, H 3.80, N, 5.27%.

### Refinement

H1NA, H2NA, H1NB and H2NB were located in a difference Fourier map and allowed to refined freely. The remaining H atoms were positioned geometrically and refined using a riding model, with C—H = 0.93 Å and *U*_iso_(H) = 1.2*U*_eq_(C). The highest residual electron density peak is located at 0.60 Å from H22A and the deepest hole is located at 0.64 Å from Sn1A.

### Crystal data

**Table:**

[Sn(C_6_H_5_)_3_(C_7_H_5_N_2_O_4_)]	*Z* = 4
*M**_r_* = 531.12	*F*(000) = 1064
Triclinic, *P*1	*D*_x_ = 1.569 Mg m^−^^3^
Hall symbol: -P 1	Mo *K*α radiation, λ = 0.71073 Å
*a* = 11.2836 (1) Å	Cell parameters from 9889 reflections
*b* = 14.9600 (2) Å	θ = 2.5–30.3°
*c* = 15.1828 (3) Å	µ = 1.17 mm^−^^1^
α = 109.257 (1)°	*T* = 296 K
β = 98.503 (1)°	Block, yellow
γ = 105.743 (1)°	0.44 × 0.32 × 0.19 mm
*V* = 2247.89 (6) Å^3^	

### Data collection

**Table:**

Bruker SMART APEXII CCD area-detector diffractometer	18711 independent reflections
Radiation source: fine-focus sealed tube	12707 reflections with *I* > 2σ(*I*)
graphite	*R*_int_ = 0.027
φ and ω scans	θ_max_ = 34.5°, θ_min_ = 1.9°
Absorption correction: multi-scan (*SADABS*; Bruker, 2009)	*h* = −17→17
*T*_min_ = 0.628, *T*_max_ = 0.810	*k* = −23→23
69259 measured reflections	*l* = −24→24

### Refinement

**Table:**

Refinement on *F*^2^	Primary atom site location: structure-invariant direct methods
Least-squares matrix: full	Secondary atom site location: difference Fourier map
*R*[*F*^2^ > 2σ(*F*^2^)] = 0.032	Hydrogen site location: inferred from neighbouring sites
*wR*(*F*^2^) = 0.080	H atoms treated by a mixture of independent and constrained refinement
*S* = 1.01	*w* = 1/[σ^2^(*F*_o_^2^) + (0.0314*P*)^2^ + 0.4012*P*] where *P* = (*F*_o_^2^ + 2*F*_c_^2^)/3
18711 reflections	(Δ/σ)_max_ = 0.003
593 parameters	Δρ_max_ = 0.70 e Å^−^^3^
0 restraints	Δρ_min_ = −0.42 e Å^−^^3^

### Special details

**Table:**

Geometry. All esds (except the esd in the dihedral angle between two l.s. planes) are estimated using the full covariance matrix. The cell esds are taken into account individually in the estimation of esds in distances, angles and torsion angles; correlations between esds in cell parameters are only used when they are defined by crystal symmetry. An approximate (isotropic) treatment of cell esds is used for estimating esds involving l.s. planes.
Refinement. Refinement of F^2^ against ALL reflections. The weighted R-factor wR and goodness of fit S are based on F^2^, conventional R-factors R are based on F, with F set to zero for negative F^2^. The threshold expression of F^2^ > 2sigma(F^2^) is used only for calculating R-factors(gt) etc. and is not relevant to the choice of reflections for refinement. R-factors based on F^2^ are statistically about twice as large as those based on F, and R- factors based on ALL data will be even larger.

### Fractional atomic coordinates and isotropic or equivalent isotropic displacement parameters (Å^2^)

**Table:**

	*x*	*y*	*z*	*U*_iso_*/*U*_eq_	
Sn1A	0.345584 (12)	0.684458 (9)	0.766833 (10)	0.05415 (4)	
N1A	0.5711 (2)	0.41300 (17)	0.82572 (15)	0.0679 (5)	
N2A	0.52199 (18)	0.26757 (13)	0.91798 (13)	0.0650 (4)	
O1A	0.33579 (15)	0.58591 (11)	0.83593 (12)	0.0692 (4)	
O2A	0.49933 (15)	0.55427 (12)	0.78536 (12)	0.0731 (4)	
O3A	0.60941 (19)	0.26953 (15)	0.87753 (15)	0.0931 (6)	
O4A	0.49988 (17)	0.21485 (14)	0.96419 (14)	0.0900 (5)	
C1A	0.09780 (18)	0.73884 (14)	0.75590 (15)	0.0555 (4)	
H1AA	0.1046	0.7370	0.6951	0.067*	
C2A	−0.0055 (2)	0.75611 (17)	0.78786 (18)	0.0670 (5)	
H2AA	−0.0685	0.7647	0.7478	0.080*	
C3A	−0.0165 (2)	0.76071 (17)	0.87803 (18)	0.0678 (5)	
H3AA	−0.0864	0.7723	0.8989	0.081*	
C4A	0.0762 (2)	0.74820 (16)	0.93671 (16)	0.0650 (5)	
H4AA	0.0698	0.7522	0.9982	0.078*	
C5A	0.1789 (2)	0.72970 (15)	0.90574 (15)	0.0607 (5)	
H5AA	0.2408	0.7207	0.9464	0.073*	
C6A	0.19167 (16)	0.72423 (13)	0.81431 (14)	0.0510 (4)	
C7A	0.30532 (19)	0.59320 (14)	0.61907 (15)	0.0586 (5)	
C8A	0.3937 (3)	0.6018 (2)	0.5665 (2)	0.0912 (8)	
H8AA	0.4755	0.6485	0.5973	0.109*	
C9A	0.3648 (3)	0.5436 (3)	0.4697 (2)	0.1051 (10)	
H9AA	0.4272	0.5509	0.4364	0.126*	
C10A	0.2474 (3)	0.4762 (2)	0.4227 (2)	0.0995 (9)	
H10A	0.2277	0.4385	0.3567	0.119*	
C11A	0.1570 (3)	0.4635 (2)	0.4728 (3)	0.1121 (11)	
H11A	0.0764	0.4152	0.4413	0.135*	
C12A	0.1854 (2)	0.5225 (2)	0.5701 (2)	0.0866 (7)	
H12A	0.1228	0.5144	0.6032	0.104*	
C13A	0.51909 (16)	0.80878 (13)	0.82951 (13)	0.0480 (4)	
C14A	0.63718 (18)	0.80336 (15)	0.81823 (16)	0.0600 (5)	
H14A	0.6447	0.7411	0.7867	0.072*	
C15A	0.74387 (19)	0.88986 (17)	0.85355 (17)	0.0671 (5)	
H15A	0.8223	0.8858	0.8444	0.080*	
C16A	0.7335 (2)	0.98195 (16)	0.90228 (14)	0.0627 (5)	
H16A	0.8055	1.0399	0.9270	0.075*	
C17A	0.6184 (2)	0.98888 (15)	0.91460 (13)	0.0591 (5)	
H17A	0.6121	1.0513	0.9473	0.071*	
C18A	0.51119 (18)	0.90262 (14)	0.87819 (13)	0.0520 (4)	
H18A	0.4329	0.9076	0.8865	0.062*	
C19A	0.4145 (2)	0.53623 (14)	0.82546 (15)	0.0575 (4)	
C20A	0.39134 (18)	0.45685 (12)	0.86679 (13)	0.0508 (4)	
C21A	0.2883 (2)	0.44010 (15)	0.90545 (15)	0.0613 (5)	
H21A	0.2350	0.4776	0.9041	0.074*	
C22A	0.2616 (2)	0.36870 (17)	0.94646 (18)	0.0712 (6)	
H22A	0.1907	0.3579	0.9712	0.085*	
C23A	0.3406 (2)	0.31507 (16)	0.94982 (16)	0.0640 (5)	
H23A	0.3249	0.2686	0.9788	0.077*	
C24A	0.44437 (18)	0.32867 (13)	0.91065 (13)	0.0528 (4)	
C25A	0.47379 (17)	0.39891 (13)	0.86597 (12)	0.0495 (4)	
Sn1B	0.018573 (11)	0.827628 (9)	0.339100 (8)	0.04415 (4)	
N1B	0.45431 (19)	1.04371 (15)	0.31356 (12)	0.0643 (5)	
N2B	0.69475 (15)	1.19985 (12)	0.43518 (13)	0.0582 (4)	
O1B	0.17982 (11)	0.93658 (9)	0.44468 (9)	0.0506 (3)	
O2B	0.22688 (11)	0.92890 (10)	0.30778 (9)	0.0547 (3)	
O3B	0.67725 (15)	1.17694 (12)	0.34679 (12)	0.0733 (4)	
O4B	0.79777 (14)	1.25610 (13)	0.49218 (13)	0.0851 (5)	
C1B	−0.2120 (2)	0.72477 (18)	0.39784 (16)	0.0722 (6)	
H1BA	−0.2403	0.6851	0.3320	0.087*	
C2B	−0.2892 (2)	0.7087 (2)	0.4578 (2)	0.0926 (8)	
H2BA	−0.3688	0.6581	0.4322	0.111*	
C3B	−0.2494 (3)	0.7666 (2)	0.5542 (2)	0.0815 (7)	
H3BA	−0.3016	0.7556	0.5944	0.098*	
C4B	−0.1336 (3)	0.83997 (18)	0.59140 (16)	0.0728 (6)	
H4BA	−0.1065	0.8797	0.6572	0.087*	
C5B	−0.0555 (2)	0.85649 (15)	0.53250 (14)	0.0596 (5)	
H5BA	0.0240	0.9072	0.5593	0.072*	
C6B	−0.09302 (16)	0.79919 (13)	0.43473 (12)	0.0462 (4)	
C7B	−0.07386 (16)	0.89100 (13)	0.25507 (12)	0.0465 (4)	
C8B	−0.03269 (19)	0.91179 (15)	0.18003 (14)	0.0569 (4)	
H8BA	0.0400	0.8994	0.1654	0.068*	
C9B	−0.0984 (2)	0.95064 (18)	0.12670 (17)	0.0717 (6)	
H9BA	−0.0698	0.9643	0.0765	0.086*	
C10B	−0.2058 (3)	0.96913 (19)	0.14761 (19)	0.0804 (7)	
H10B	−0.2495	0.9957	0.1120	0.097*	
C11B	−0.2486 (2)	0.9484 (2)	0.22107 (18)	0.0805 (7)	
H11B	−0.3212	0.9611	0.2355	0.097*	
C12B	−0.1834 (2)	0.90832 (17)	0.27374 (15)	0.0629 (5)	
H12B	−0.2140	0.8929	0.3225	0.076*	
C13B	0.05632 (15)	0.69726 (13)	0.25895 (12)	0.0457 (3)	
C14B	0.02836 (19)	0.61415 (15)	0.28369 (15)	0.0599 (5)	
H14B	−0.0008	0.6174	0.3385	0.072*	
C15B	0.0436 (2)	0.52591 (16)	0.2271 (2)	0.0773 (7)	
H15B	0.0249	0.4704	0.2443	0.093*	
C16B	0.0857 (2)	0.52049 (17)	0.1467 (2)	0.0803 (7)	
H16B	0.0950	0.4610	0.1088	0.096*	
C17B	0.1141 (2)	0.60122 (18)	0.12143 (17)	0.0755 (6)	
H17B	0.1424	0.5968	0.0662	0.091*	
C18B	0.1010 (2)	0.68972 (15)	0.17743 (14)	0.0591 (5)	
H18B	0.1224	0.7451	0.1603	0.071*	
C19B	0.25866 (15)	0.96723 (13)	0.39734 (13)	0.0452 (3)	
C20B	0.38243 (15)	1.04711 (12)	0.45527 (11)	0.0406 (3)	
C21B	0.40676 (17)	1.08618 (14)	0.55438 (13)	0.0503 (4)	
H21B	0.3454	1.0604	0.5827	0.060*	
C22B	0.5194 (2)	1.16257 (15)	0.61373 (13)	0.0596 (5)	
H22B	0.5324	1.1885	0.6805	0.071*	
C23B	0.61088 (18)	1.19894 (14)	0.57229 (14)	0.0550 (4)	
H23B	0.6870	1.2499	0.6111	0.066*	
C24B	0.59078 (15)	1.16028 (12)	0.47263 (13)	0.0462 (4)	
C25B	0.47582 (15)	1.08341 (12)	0.40975 (12)	0.0422 (3)	
H1NA	0.622 (2)	0.382 (2)	0.8298 (18)	0.082 (8)*	
H2NA	0.584 (2)	0.459 (2)	0.8047 (18)	0.080 (8)*	
H1NB	0.385 (2)	0.9977 (18)	0.2799 (16)	0.066 (7)*	
H2NB	0.509 (2)	1.0718 (16)	0.2916 (15)	0.063 (6)*	

### Atomic displacement parameters (Å^2^)

**Table:**

	*U*^11^	*U*^22^	*U*^33^	*U*^12^	*U*^13^	*U*^23^
Sn1A	0.04460 (7)	0.04622 (7)	0.07074 (9)	0.01421 (5)	0.01363 (6)	0.02335 (6)
N1A	0.0716 (12)	0.0704 (12)	0.0796 (13)	0.0335 (10)	0.0282 (10)	0.0402 (11)
N2A	0.0665 (11)	0.0587 (10)	0.0643 (10)	0.0175 (8)	0.0010 (8)	0.0274 (9)
O1A	0.0741 (9)	0.0562 (8)	0.0905 (11)	0.0309 (7)	0.0241 (8)	0.0365 (8)
O2A	0.0750 (10)	0.0716 (10)	0.0917 (11)	0.0294 (8)	0.0286 (9)	0.0484 (9)
O3A	0.1010 (14)	0.1063 (14)	0.1205 (15)	0.0650 (12)	0.0494 (12)	0.0708 (13)
O4A	0.0896 (12)	0.0917 (12)	0.1088 (13)	0.0313 (10)	0.0143 (10)	0.0683 (11)
C1A	0.0564 (11)	0.0529 (10)	0.0639 (11)	0.0192 (8)	0.0167 (9)	0.0302 (9)
C2A	0.0607 (12)	0.0718 (13)	0.0852 (15)	0.0347 (11)	0.0190 (11)	0.0412 (12)
C3A	0.0678 (13)	0.0641 (13)	0.0845 (15)	0.0323 (11)	0.0323 (12)	0.0315 (12)
C4A	0.0771 (14)	0.0608 (12)	0.0606 (12)	0.0258 (11)	0.0228 (10)	0.0240 (10)
C5A	0.0595 (11)	0.0576 (11)	0.0607 (11)	0.0189 (9)	0.0045 (9)	0.0235 (9)
C6A	0.0430 (9)	0.0398 (8)	0.0662 (11)	0.0093 (7)	0.0097 (8)	0.0215 (8)
C7A	0.0532 (10)	0.0487 (10)	0.0720 (12)	0.0168 (8)	0.0107 (9)	0.0241 (9)
C8A	0.0702 (15)	0.0901 (19)	0.0861 (18)	0.0035 (13)	0.0237 (13)	0.0188 (15)
C9A	0.105 (2)	0.109 (2)	0.089 (2)	0.0229 (19)	0.0405 (18)	0.0291 (18)
C10A	0.117 (3)	0.093 (2)	0.0711 (16)	0.0404 (19)	0.0080 (17)	0.0138 (15)
C11A	0.087 (2)	0.094 (2)	0.101 (2)	0.0076 (17)	−0.0074 (18)	0.0046 (18)
C12A	0.0650 (14)	0.0775 (16)	0.0903 (18)	0.0084 (12)	0.0136 (13)	0.0154 (14)
C13A	0.0468 (9)	0.0504 (9)	0.0501 (9)	0.0154 (7)	0.0118 (7)	0.0248 (8)
C14A	0.0505 (10)	0.0541 (10)	0.0772 (13)	0.0215 (8)	0.0154 (9)	0.0254 (10)
C15A	0.0438 (10)	0.0728 (14)	0.0842 (15)	0.0154 (9)	0.0140 (10)	0.0349 (12)
C16A	0.0641 (12)	0.0563 (11)	0.0506 (10)	0.0022 (9)	0.0057 (9)	0.0186 (9)
C17A	0.0783 (13)	0.0508 (10)	0.0428 (9)	0.0154 (9)	0.0208 (9)	0.0146 (8)
C18A	0.0562 (10)	0.0573 (10)	0.0477 (9)	0.0210 (8)	0.0223 (8)	0.0218 (8)
C19A	0.0594 (11)	0.0462 (9)	0.0598 (11)	0.0153 (8)	0.0062 (9)	0.0180 (9)
C20A	0.0550 (10)	0.0380 (8)	0.0481 (9)	0.0116 (7)	0.0031 (7)	0.0108 (7)
C21A	0.0586 (11)	0.0516 (10)	0.0673 (12)	0.0148 (9)	0.0143 (9)	0.0194 (9)
C22A	0.0671 (13)	0.0625 (12)	0.0832 (15)	0.0159 (10)	0.0289 (12)	0.0286 (12)
C23A	0.0665 (13)	0.0533 (11)	0.0653 (12)	0.0091 (9)	0.0128 (10)	0.0258 (10)
C24A	0.0552 (10)	0.0432 (9)	0.0487 (9)	0.0119 (8)	0.0001 (8)	0.0139 (7)
C25A	0.0501 (9)	0.0426 (8)	0.0443 (8)	0.0091 (7)	0.0030 (7)	0.0121 (7)
Sn1B	0.04191 (6)	0.04688 (6)	0.04199 (6)	0.01176 (5)	0.01300 (4)	0.01712 (5)
N1B	0.0568 (10)	0.0749 (12)	0.0463 (8)	−0.0049 (9)	0.0088 (8)	0.0296 (9)
N2B	0.0469 (8)	0.0537 (9)	0.0721 (11)	0.0078 (7)	0.0127 (8)	0.0307 (8)
O1B	0.0409 (6)	0.0520 (7)	0.0567 (7)	0.0088 (5)	0.0139 (5)	0.0237 (6)
O2B	0.0441 (6)	0.0573 (7)	0.0501 (7)	0.0051 (5)	0.0039 (5)	0.0191 (6)
O3B	0.0664 (9)	0.0745 (10)	0.0738 (10)	0.0054 (7)	0.0280 (8)	0.0334 (8)
O4B	0.0477 (8)	0.0886 (12)	0.0962 (12)	−0.0068 (8)	0.0047 (8)	0.0382 (10)
C1B	0.0516 (11)	0.0810 (15)	0.0603 (12)	−0.0035 (10)	0.0121 (9)	0.0206 (11)
C2B	0.0570 (13)	0.106 (2)	0.110 (2)	0.0019 (13)	0.0320 (14)	0.0519 (18)
C3B	0.0943 (18)	0.0956 (18)	0.0986 (19)	0.0467 (15)	0.0634 (16)	0.0639 (16)
C4B	0.1108 (19)	0.0692 (14)	0.0558 (11)	0.0375 (14)	0.0408 (12)	0.0323 (11)
C5B	0.0695 (12)	0.0515 (10)	0.0490 (10)	0.0098 (9)	0.0152 (9)	0.0177 (8)
C6B	0.0458 (9)	0.0463 (8)	0.0452 (8)	0.0118 (7)	0.0145 (7)	0.0184 (7)
C7B	0.0457 (9)	0.0436 (8)	0.0424 (8)	0.0122 (7)	0.0069 (7)	0.0117 (7)
C8B	0.0510 (10)	0.0642 (12)	0.0590 (11)	0.0178 (9)	0.0134 (8)	0.0302 (10)
C9B	0.0705 (14)	0.0792 (15)	0.0685 (13)	0.0192 (12)	0.0091 (11)	0.0418 (12)
C10B	0.0841 (16)	0.0780 (15)	0.0809 (16)	0.0365 (13)	−0.0002 (13)	0.0353 (13)
C11B	0.0751 (15)	0.0960 (18)	0.0774 (15)	0.0518 (14)	0.0147 (12)	0.0265 (14)
C12B	0.0605 (12)	0.0768 (14)	0.0538 (10)	0.0327 (10)	0.0177 (9)	0.0196 (10)
C13B	0.0403 (8)	0.0457 (8)	0.0462 (8)	0.0109 (7)	0.0081 (7)	0.0163 (7)
C14B	0.0606 (11)	0.0508 (10)	0.0624 (11)	0.0087 (9)	0.0162 (9)	0.0231 (9)
C15B	0.0817 (16)	0.0453 (11)	0.0990 (18)	0.0122 (10)	0.0216 (14)	0.0294 (12)
C16B	0.0798 (16)	0.0501 (12)	0.0991 (18)	0.0207 (11)	0.0317 (14)	0.0121 (12)
C17B	0.0826 (15)	0.0662 (14)	0.0705 (14)	0.0234 (12)	0.0355 (12)	0.0127 (11)
C18B	0.0707 (12)	0.0551 (11)	0.0549 (10)	0.0219 (9)	0.0234 (9)	0.0224 (9)
C19B	0.0397 (8)	0.0447 (8)	0.0555 (9)	0.0153 (7)	0.0113 (7)	0.0246 (8)
C20B	0.0375 (7)	0.0405 (8)	0.0454 (8)	0.0138 (6)	0.0090 (6)	0.0191 (7)
C21B	0.0498 (9)	0.0523 (10)	0.0489 (9)	0.0176 (8)	0.0171 (7)	0.0177 (8)
C22B	0.0642 (12)	0.0592 (11)	0.0425 (9)	0.0170 (9)	0.0089 (8)	0.0095 (8)
C23B	0.0504 (10)	0.0468 (9)	0.0532 (10)	0.0100 (8)	0.0002 (8)	0.0124 (8)
C24B	0.0399 (8)	0.0424 (8)	0.0559 (9)	0.0106 (6)	0.0081 (7)	0.0234 (7)
C25B	0.0419 (8)	0.0427 (8)	0.0446 (8)	0.0126 (6)	0.0078 (6)	0.0232 (7)

### Geometric parameters (Å, °)

**Table:**

Sn1A—O1A	2.0621 (14)	Sn1B—O1B	2.0836 (12)
Sn1A—C7A	2.108 (2)	Sn1B—C13B	2.1224 (17)
Sn1A—C13A	2.1221 (18)	Sn1B—C6B	2.1289 (16)
Sn1A—C6A	2.1302 (19)	Sn1B—C7B	2.1343 (17)
N1A—C25A	1.334 (3)	N1B—C25B	1.338 (2)
N1A—H1NA	0.84 (3)	N1B—H1NB	0.84 (2)
N1A—H2NA	0.84 (3)	N1B—H2NB	0.83 (2)
N2A—O4A	1.219 (2)	N2B—O4B	1.225 (2)
N2A—O3A	1.235 (2)	N2B—O3B	1.240 (2)
N2A—C24A	1.445 (3)	N2B—C24B	1.447 (2)
O1A—C19A	1.300 (2)	O1B—C19B	1.305 (2)
O2A—C19A	1.225 (3)	O2B—C19B	1.239 (2)
C1A—C2A	1.382 (3)	C1B—C6B	1.382 (3)
C1A—C6A	1.390 (3)	C1B—C2B	1.383 (3)
C1A—H1AA	0.9300	C1B—H1BA	0.9300
C2A—C3A	1.374 (3)	C2B—C3B	1.361 (4)
C2A—H2AA	0.9300	C2B—H2BA	0.9300
C3A—C4A	1.365 (3)	C3B—C4B	1.351 (4)
C3A—H3AA	0.9300	C3B—H3BA	0.9300
C4A—C5A	1.374 (3)	C4B—C5B	1.378 (3)
C4A—H4AA	0.9300	C4B—H4BA	0.9300
C5A—C6A	1.395 (3)	C5B—C6B	1.377 (2)
C5A—H5AA	0.9300	C5B—H5BA	0.9300
C7A—C8A	1.371 (3)	C7B—C12B	1.380 (3)
C7A—C12A	1.382 (3)	C7B—C8B	1.387 (3)
C8A—C9A	1.373 (4)	C8B—C9B	1.382 (3)
C8A—H8AA	0.9300	C8B—H8BA	0.9300
C9A—C10A	1.342 (4)	C9B—C10B	1.372 (3)
C9A—H9AA	0.9300	C9B—H9BA	0.9300
C10A—C11A	1.370 (5)	C10B—C11B	1.371 (4)
C10A—H10A	0.9300	C10B—H10B	0.9300
C11A—C12A	1.383 (4)	C11B—C12B	1.386 (3)
C11A—H11A	0.9300	C11B—H11B	0.9300
C12A—H12A	0.9300	C12B—H12B	0.9300
C13A—C18A	1.388 (3)	C13B—C14B	1.384 (3)
C13A—C14A	1.389 (3)	C13B—C18B	1.386 (2)
C14A—C15A	1.385 (3)	C14B—C15B	1.389 (3)
C14A—H14A	0.9300	C14B—H14B	0.9300
C15A—C16A	1.378 (3)	C15B—C16B	1.361 (4)
C15A—H15A	0.9300	C15B—H15B	0.9300
C16A—C17A	1.365 (3)	C16B—C17B	1.357 (3)
C16A—H16A	0.9300	C16B—H16B	0.9300
C17A—C18A	1.386 (3)	C17B—C18B	1.378 (3)
C17A—H17A	0.9300	C17B—H17B	0.9300
C18A—H18A	0.9300	C18B—H18B	0.9300
C19A—C20A	1.498 (3)	C19B—C20B	1.480 (2)
C20A—C21A	1.378 (3)	C20B—C21B	1.375 (2)
C20A—C25A	1.432 (3)	C20B—C25B	1.427 (2)
C21A—C22A	1.392 (3)	C21B—C22B	1.388 (3)
C21A—H21A	0.9300	C21B—H21B	0.9300
C22A—C23A	1.358 (3)	C22B—C23B	1.368 (3)
C22A—H22A	0.9300	C22B—H22B	0.9300
C23A—C24A	1.387 (3)	C23B—C24B	1.388 (3)
C23A—H23A	0.9300	C23B—H23B	0.9300
C24A—C25A	1.422 (3)	C24B—C25B	1.420 (2)
			
O1A—Sn1A—C7A	104.63 (7)	O1B—Sn1B—C13B	112.17 (6)
O1A—Sn1A—C13A	111.31 (6)	O1B—Sn1B—C6B	96.96 (6)
C7A—Sn1A—C13A	117.86 (7)	C13B—Sn1B—C6B	111.36 (7)
O1A—Sn1A—C6A	92.35 (6)	O1B—Sn1B—C7B	111.49 (6)
C7A—Sn1A—C6A	116.06 (7)	C13B—Sn1B—C7B	115.50 (6)
C13A—Sn1A—C6A	111.28 (7)	C6B—Sn1B—C7B	107.75 (7)
C25A—N1A—H1NA	119.3 (18)	C25B—N1B—H1NB	119.7 (15)
C25A—N1A—H2NA	117.2 (17)	C25B—N1B—H2NB	115.4 (15)
H1NA—N1A—H2NA	123 (3)	H1NB—N1B—H2NB	125 (2)
O4A—N2A—O3A	121.1 (2)	O4B—N2B—O3B	121.77 (17)
O4A—N2A—C24A	119.2 (2)	O4B—N2B—C24B	118.73 (17)
O3A—N2A—C24A	119.66 (18)	O3B—N2B—C24B	119.49 (15)
C19A—O1A—Sn1A	116.68 (14)	C19B—O1B—Sn1B	105.37 (10)
C2A—C1A—C6A	120.27 (19)	C6B—C1B—C2B	120.7 (2)
C2A—C1A—H1AA	119.9	C6B—C1B—H1BA	119.7
C6A—C1A—H1AA	119.9	C2B—C1B—H1BA	119.7
C3A—C2A—C1A	120.9 (2)	C3B—C2B—C1B	120.4 (2)
C3A—C2A—H2AA	119.6	C3B—C2B—H2BA	119.8
C1A—C2A—H2AA	119.6	C1B—C2B—H2BA	119.8
C4A—C3A—C2A	119.4 (2)	C4B—C3B—C2B	119.7 (2)
C4A—C3A—H3AA	120.3	C4B—C3B—H3BA	120.1
C2A—C3A—H3AA	120.3	C2B—C3B—H3BA	120.1
C3A—C4A—C5A	120.6 (2)	C3B—C4B—C5B	120.5 (2)
C3A—C4A—H4AA	119.7	C3B—C4B—H4BA	119.7
C5A—C4A—H4AA	119.7	C5B—C4B—H4BA	119.7
C4A—C5A—C6A	121.00 (19)	C6B—C5B—C4B	121.2 (2)
C4A—C5A—H5AA	119.5	C6B—C5B—H5BA	119.4
C6A—C5A—H5AA	119.5	C4B—C5B—H5BA	119.4
C1A—C6A—C5A	117.86 (18)	C5B—C6B—C1B	117.59 (17)
C1A—C6A—Sn1A	122.98 (15)	C5B—C6B—Sn1B	122.68 (13)
C5A—C6A—Sn1A	119.03 (14)	C1B—C6B—Sn1B	119.60 (14)
C8A—C7A—C12A	116.9 (2)	C12B—C7B—C8B	118.21 (17)
C8A—C7A—Sn1A	122.62 (17)	C12B—C7B—Sn1B	118.28 (14)
C12A—C7A—Sn1A	120.47 (18)	C8B—C7B—Sn1B	123.46 (14)
C7A—C8A—C9A	121.8 (3)	C9B—C8B—C7B	120.8 (2)
C7A—C8A—H8AA	119.1	C9B—C8B—H8BA	119.6
C9A—C8A—H8AA	119.1	C7B—C8B—H8BA	119.6
C10A—C9A—C8A	120.7 (3)	C10B—C9B—C8B	120.1 (2)
C10A—C9A—H9AA	119.6	C10B—C9B—H9BA	119.9
C8A—C9A—H9AA	119.6	C8B—C9B—H9BA	119.9
C9A—C10A—C11A	119.4 (3)	C11B—C10B—C9B	120.0 (2)
C9A—C10A—H10A	120.3	C11B—C10B—H10B	120.0
C11A—C10A—H10A	120.3	C9B—C10B—H10B	120.0
C10A—C11A—C12A	120.0 (3)	C10B—C11B—C12B	119.8 (2)
C10A—C11A—H11A	120.0	C10B—C11B—H11B	120.1
C12A—C11A—H11A	120.0	C12B—C11B—H11B	120.1
C7A—C12A—C11A	121.1 (3)	C7B—C12B—C11B	121.1 (2)
C7A—C12A—H12A	119.5	C7B—C12B—H12B	119.5
C11A—C12A—H12A	119.5	C11B—C12B—H12B	119.5
C18A—C13A—C14A	118.41 (17)	C14B—C13B—C18B	118.06 (17)
C18A—C13A—Sn1A	117.13 (13)	C14B—C13B—Sn1B	120.07 (14)
C14A—C13A—Sn1A	124.24 (14)	C18B—C13B—Sn1B	121.73 (13)
C15A—C14A—C13A	120.58 (19)	C13B—C14B—C15B	120.3 (2)
C15A—C14A—H14A	119.7	C13B—C14B—H14B	119.8
C13A—C14A—H14A	119.7	C15B—C14B—H14B	119.8
C16A—C15A—C14A	119.8 (2)	C16B—C15B—C14B	120.1 (2)
C16A—C15A—H15A	120.1	C16B—C15B—H15B	119.9
C14A—C15A—H15A	120.1	C14B—C15B—H15B	119.9
C17A—C16A—C15A	120.56 (19)	C17B—C16B—C15B	120.4 (2)
C17A—C16A—H16A	119.7	C17B—C16B—H16B	119.8
C15A—C16A—H16A	119.7	C15B—C16B—H16B	119.8
C16A—C17A—C18A	119.78 (19)	C16B—C17B—C18B	120.2 (2)
C16A—C17A—H17A	120.1	C16B—C17B—H17B	119.9
C18A—C17A—H17A	120.1	C18B—C17B—H17B	119.9
C17A—C18A—C13A	120.87 (18)	C17B—C18B—C13B	120.86 (19)
C17A—C18A—H18A	119.6	C17B—C18B—H18B	119.6
C13A—C18A—H18A	119.6	C13B—C18B—H18B	119.6
O2A—C19A—O1A	121.92 (19)	O2B—C19B—O1B	119.18 (15)
O2A—C19A—C20A	124.23 (19)	O2B—C19B—C20B	123.75 (15)
O1A—C19A—C20A	113.85 (18)	O1B—C19B—C20B	117.07 (15)
C21A—C20A—C25A	120.37 (18)	C21B—C20B—C25B	120.08 (15)
C21A—C20A—C19A	118.65 (18)	C21B—C20B—C19B	118.97 (15)
C25A—C20A—C19A	120.98 (17)	C25B—C20B—C19B	120.95 (14)
C20A—C21A—C22A	122.0 (2)	C20B—C21B—C22B	122.50 (17)
C20A—C21A—H21A	119.0	C20B—C21B—H21B	118.8
C22A—C21A—H21A	119.0	C22B—C21B—H21B	118.8
C23A—C22A—C21A	119.0 (2)	C23B—C22B—C21B	118.82 (17)
C23A—C22A—H22A	120.5	C23B—C22B—H22B	120.6
C21A—C22A—H22A	120.5	C21B—C22B—H22B	120.6
C22A—C23A—C24A	120.8 (2)	C22B—C23B—C24B	120.39 (17)
C22A—C23A—H23A	119.6	C22B—C23B—H23B	119.8
C24A—C23A—H23A	119.6	C24B—C23B—H23B	119.8
C23A—C24A—C25A	122.25 (18)	C23B—C24B—C25B	122.25 (16)
C23A—C24A—N2A	116.25 (18)	C23B—C24B—N2B	116.73 (15)
C25A—C24A—N2A	121.49 (18)	C25B—C24B—N2B	120.99 (16)
N1A—C25A—C24A	124.23 (19)	N1B—C25B—C24B	123.63 (16)
N1A—C25A—C20A	120.28 (18)	N1B—C25B—C20B	120.39 (16)
C24A—C25A—C20A	115.49 (17)	C24B—C25B—C20B	115.95 (15)
			
C7A—Sn1A—O1A—C19A	60.17 (16)	C13B—Sn1B—O1B—C19B	66.64 (12)
C13A—Sn1A—O1A—C19A	−68.16 (16)	C6B—Sn1B—O1B—C19B	−176.88 (11)
C6A—Sn1A—O1A—C19A	177.90 (15)	C7B—Sn1B—O1B—C19B	−64.66 (12)
C6A—C1A—C2A—C3A	1.0 (3)	C6B—C1B—C2B—C3B	0.4 (4)
C1A—C2A—C3A—C4A	0.1 (3)	C1B—C2B—C3B—C4B	−0.1 (4)
C2A—C3A—C4A—C5A	−0.9 (3)	C2B—C3B—C4B—C5B	−0.2 (4)
C3A—C4A—C5A—C6A	0.6 (3)	C3B—C4B—C5B—C6B	0.1 (4)
C2A—C1A—C6A—C5A	−1.3 (3)	C4B—C5B—C6B—C1B	0.2 (3)
C2A—C1A—C6A—Sn1A	174.47 (15)	C4B—C5B—C6B—Sn1B	175.92 (16)
C4A—C5A—C6A—C1A	0.5 (3)	C2B—C1B—C6B—C5B	−0.5 (4)
C4A—C5A—C6A—Sn1A	−175.45 (15)	C2B—C1B—C6B—Sn1B	−176.3 (2)
O1A—Sn1A—C6A—C1A	−140.20 (15)	O1B—Sn1B—C6B—C5B	7.96 (17)
C7A—Sn1A—C6A—C1A	−32.66 (17)	C13B—Sn1B—C6B—C5B	125.08 (16)
C13A—Sn1A—C6A—C1A	105.83 (15)	C7B—Sn1B—C6B—C5B	−107.29 (16)
O1A—Sn1A—C6A—C5A	35.51 (15)	O1B—Sn1B—C6B—C1B	−176.38 (17)
C7A—Sn1A—C6A—C5A	143.05 (14)	C13B—Sn1B—C6B—C1B	−59.26 (18)
C13A—Sn1A—C6A—C5A	−78.46 (15)	C7B—Sn1B—C6B—C1B	68.37 (18)
O1A—Sn1A—C7A—C8A	−114.3 (2)	O1B—Sn1B—C7B—C12B	−102.93 (15)
C13A—Sn1A—C7A—C8A	9.9 (2)	C13B—Sn1B—C7B—C12B	127.50 (15)
C6A—Sn1A—C7A—C8A	145.6 (2)	C6B—Sn1B—C7B—C12B	2.31 (16)
O1A—Sn1A—C7A—C12A	67.1 (2)	O1B—Sn1B—C7B—C8B	79.86 (16)
C13A—Sn1A—C7A—C12A	−168.65 (18)	C13B—Sn1B—C7B—C8B	−49.71 (17)
C6A—Sn1A—C7A—C12A	−33.0 (2)	C6B—Sn1B—C7B—C8B	−174.90 (15)
C12A—C7A—C8A—C9A	0.4 (4)	C12B—C7B—C8B—C9B	1.2 (3)
Sn1A—C7A—C8A—C9A	−178.2 (2)	Sn1B—C7B—C8B—C9B	178.44 (16)
C7A—C8A—C9A—C10A	0.6 (5)	C7B—C8B—C9B—C10B	0.0 (3)
C8A—C9A—C10A—C11A	−2.0 (6)	C8B—C9B—C10B—C11B	−0.5 (4)
C9A—C10A—C11A—C12A	2.5 (6)	C9B—C10B—C11B—C12B	−0.3 (4)
C8A—C7A—C12A—C11A	0.1 (4)	C8B—C7B—C12B—C11B	−2.0 (3)
Sn1A—C7A—C12A—C11A	178.7 (2)	Sn1B—C7B—C12B—C11B	−179.35 (18)
C10A—C11A—C12A—C7A	−1.5 (5)	C10B—C11B—C12B—C7B	1.5 (4)
O1A—Sn1A—C13A—C18A	−112.38 (13)	O1B—Sn1B—C13B—C14B	96.73 (15)
C7A—Sn1A—C13A—C18A	126.77 (13)	C6B—Sn1B—C13B—C14B	−10.72 (16)
C6A—Sn1A—C13A—C18A	−10.89 (15)	C7B—Sn1B—C13B—C14B	−134.04 (14)
O1A—Sn1A—C13A—C14A	73.02 (17)	O1B—Sn1B—C13B—C18B	−87.64 (15)
C7A—Sn1A—C13A—C14A	−47.83 (18)	C6B—Sn1B—C13B—C18B	164.91 (14)
C6A—Sn1A—C13A—C14A	174.51 (15)	C7B—Sn1B—C13B—C18B	41.60 (17)
C18A—C13A—C14A—C15A	−0.9 (3)	C18B—C13B—C14B—C15B	−0.8 (3)
Sn1A—C13A—C14A—C15A	173.58 (16)	Sn1B—C13B—C14B—C15B	175.01 (17)
C13A—C14A—C15A—C16A	1.5 (3)	C13B—C14B—C15B—C16B	−0.3 (4)
C14A—C15A—C16A—C17A	−1.1 (3)	C14B—C15B—C16B—C17B	0.5 (4)
C15A—C16A—C17A—C18A	0.3 (3)	C15B—C16B—C17B—C18B	0.3 (4)
C16A—C17A—C18A—C13A	0.3 (3)	C16B—C17B—C18B—C13B	−1.4 (4)
C14A—C13A—C18A—C17A	0.1 (3)	C14B—C13B—C18B—C17B	1.6 (3)
Sn1A—C13A—C18A—C17A	−174.86 (14)	Sn1B—C13B—C18B—C17B	−174.13 (17)
Sn1A—O1A—C19A—O2A	7.5 (3)	Sn1B—O1B—C19B—O2B	−0.83 (18)
Sn1A—O1A—C19A—C20A	−172.66 (12)	Sn1B—O1B—C19B—C20B	179.09 (11)
O2A—C19A—C20A—C21A	−176.47 (19)	O2B—C19B—C20B—C21B	178.42 (17)
O1A—C19A—C20A—C21A	3.7 (3)	O1B—C19B—C20B—C21B	−1.5 (2)
O2A—C19A—C20A—C25A	3.2 (3)	O2B—C19B—C20B—C25B	−1.7 (2)
O1A—C19A—C20A—C25A	−176.63 (16)	O1B—C19B—C20B—C25B	178.38 (14)
C25A—C20A—C21A—C22A	1.3 (3)	C25B—C20B—C21B—C22B	1.4 (3)
C19A—C20A—C21A—C22A	−179.03 (19)	C19B—C20B—C21B—C22B	−178.70 (17)
C20A—C21A—C22A—C23A	1.0 (3)	C20B—C21B—C22B—C23B	−1.4 (3)
C21A—C22A—C23A—C24A	−1.9 (3)	C21B—C22B—C23B—C24B	0.3 (3)
C22A—C23A—C24A—C25A	0.4 (3)	C22B—C23B—C24B—C25B	0.8 (3)
C22A—C23A—C24A—N2A	−179.87 (19)	C22B—C23B—C24B—N2B	−177.28 (18)
O4A—N2A—C24A—C23A	−6.1 (3)	O4B—N2B—C24B—C23B	7.7 (3)
O3A—N2A—C24A—C23A	173.9 (2)	O3B—N2B—C24B—C23B	−171.67 (18)
O4A—N2A—C24A—C25A	173.64 (18)	O4B—N2B—C24B—C25B	−170.48 (18)
O3A—N2A—C24A—C25A	−6.3 (3)	O3B—N2B—C24B—C25B	10.2 (3)
C23A—C24A—C25A—N1A	−178.63 (19)	C23B—C24B—C25B—N1B	−179.08 (18)
N2A—C24A—C25A—N1A	1.6 (3)	N2B—C24B—C25B—N1B	−1.1 (3)
C23A—C24A—C25A—C20A	1.8 (3)	C23B—C24B—C25B—C20B	−0.8 (2)
N2A—C24A—C25A—C20A	−177.91 (16)	N2B—C24B—C25B—C20B	177.20 (15)
C21A—C20A—C25A—N1A	177.84 (18)	C21B—C20B—C25B—N1B	178.04 (18)
C19A—C20A—C25A—N1A	−1.9 (3)	C19B—C20B—C25B—N1B	−1.8 (2)
C21A—C20A—C25A—C24A	−2.6 (2)	C21B—C20B—C25B—C24B	−0.3 (2)
C19A—C20A—C25A—C24A	177.70 (15)	C19B—C20B—C25B—C24B	179.85 (14)

### Hydrogen-bond geometry (Å, °)

**Table:**

*Cg*1 is the centroid of the C1A–C6A phenyl ring.

**Table:**

*D*—H···*A*	*D*—H	H···*A*	*D*···*A*	*D*—H···*A*
N1A—H1NA···O3A	0.84 (3)	2.02 (3)	2.632 (3)	129 (2)
N1A—H2NA···O2A	0.84 (3)	1.99 (3)	2.671 (3)	138 (2)
N1B—H1NB···O2B	0.84 (2)	1.98 (3)	2.643 (3)	135 (2)
N1B—H2NB···O3B	0.83 (2)	1.96 (2)	2.607 (3)	135.3 (19)
C15B—H15B···Cg1^i^	0.93	2.84	3.596 (3)	139

Symmetry codes: (i) −*x*, −*y*+1, −*z*+1.
